# Radi Macruz – O Legado de um Ícone

**DOI:** 10.36660/abc.20201341

**Published:** 2021-04-08

**Affiliations:** 1 Universidade de São Paulo Faculdade de Medicina Hospital das Clínicas São PauloSP Brasil Instituto do Coração do Hospital das Clínicas da Faculdade de Medicina da Universidade de São Paulo, São Paulo, SP - Brasil.

**Keywords:** Radi Macruz, Cardiologistas/tendências, Docentes, Cardiologia, Médicos, Ética Médica

Radi Macruz, Professor-associado do Departamento de Cardiopneumologia da Faculdade de Medicina da Universidade de São Paulo, faleceu em São Paulo em 30 de novembro de 2020, aos 95 anos.

Nasceu em 5 de julho de 1925 em São Paulo, filho dos libaneses Kalil e Nini Macruz, tornou-se, após formação acadêmica de Medicina na FMUSP, o grande médico e pioneiro de muitos avanços que o caracterizaram como um dos ícones de maior relevância. Era um idealizador, na perseguição da dinâmica mais adequada para a ciência e para as descobertas científicas.

Sua trajetória acadêmica foi calcada sempre no profundo conhecimento e no progresso da ciência cardiológica como um todo. Cursou Medicina na Universidade de São Paulo, na qual iniciou em 1946, estimulado pelo pai que salientava a importância do médico na sociedade e apesar da Matemática, sua paixão desde jovem, que melhor conhecia por seus estudos nas escolas do interior de São Paulo, onde morou com mais dez irmãos, além dos sete primos agregados de seu tio paterno.

No entanto, durante seu crescimento profissional, foi a Matemática que influenciou sua trajetória científica na Cardiologia, interligadas em muitos aspectos. Nessa dinâmica, tal fator resultou na criação do índice que leva seu nome, o do intervalo PPR do eletrocardiograma na caracterização das sobrecargas atriais.^1^^–^^4^

Certa vez, por esta criação e tantas outras mais, o Instituto do Coração (InCor) recebeu uma das maiores autoridades em eletrocardiografia do mundo, o sul-africano Leo Schamroth,^5^^,^^6^para proferir uma palestra. Conduzido pelo Dr. Charles Mady ao anfiteatro, houve o encontro com Macruz na porta do mesmo. Na apresentação de ambos, Schamroth perguntou se ele era o mesmo do índice de Macruz. Mady respondeu afirmativamente e apressou-se em abrir o seu livro e mostrar a página comentando a respeito do referido sinal. Tornaram-se amigos. Não precisava de grandes tecnologias para Macruz mostrar a sua genialidade.

**Figura 1:**
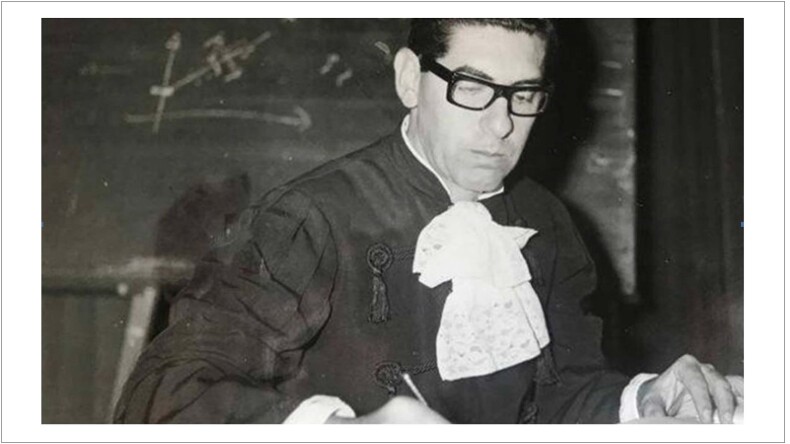
Foto do Radi Macruz, na expressão da seriedade e responsabilidade, decorrentes do pensamento concentrado em possíveis descobertas científicas.

Ele acreditava, em sua percepção, que não existe Medicina sem Humanismo, que a paixão da vida era a descoberta de diferentes coisas, que a massa crítica desenvolve o pensamento e o desenvolvimento, que a lógica atinge a verdade, que o espírito da pesquisa nada maisé do que a própria indagação, que a paixão era desvendar os mistérios da natureza (Figura 1).

Desde que voltou dos Estados Unidos, participando de pesquisas no grupo de seu amigo Sarnoff,^7^ tornou-se uma fonte inesgotável de ideias e produção científica. Como tinha uma formação geral muito ampla, não se limitou a criar subespecialidades, o que era pouco para ele, tendo bases sólidas em Matemática e estatística, algo que o ajudou muito.

Com todo esse direcionamento de vida, iniciou-se na procura do entendimento da Cardiologia Pediátrica, especialidade que suscitava muita dificuldade diagnóstica e terapêutica, principalmente no período que compreendia as décadas de 1950 a 1980. Desenvolveu-a durante este tempo, em conjunto com Munir Ebaid, tornando-se um dos responsáveis pelo reconhecimento mundial nessa especialidade e como um dos ícones brasileiros.

Inovou com o tratamento da hipertensão pulmonar em cardiopatias congênitas, através da abertura de uma comunicação interatrial. Lançou conceitos originais sobre a normalidade individual da pressão arterial. Queria realizar a transposição das grandes artérias em pacientes com insuficiência cardíaca, estando normal o ventrículo direito.

Tal era o seu conhecimento que todos o procuravam no discernimento das dificuldades de conduta. Quando iniciava suas visitas médicas, sempre havia muita afluência de pessoas interessadas, pois suas discussões à beira do leito eram antológicas e muito disputadas. Diagnosticava cardiopatias complexas na beira do leito com exame físico, radiologia simples e eletrocardiografia, simplesmente baseado no raciocínio clínico. Era um dos raros médicos que não tinha receio de expor o seu modo de pensar numa discussão clínica: muitas vezes alguém achava absurdo o que ele dizia e, mais tarde, se curvava à luz que brilhou.

Por estes motivos e com tal motivação ao adentrar a enfermaria no Hospital das Clínicas ou no Instituto do Coração, apagava as luzes e dizia que “a luz havia chegado”.

Em 1983, não de se estranhar quando lançou o primeiro livro da especialidade no Brasil, intitulado Cardiologia Pediátrica, em conjunto com a Dra. Rachel Snitcovsky, pela Editora Sarvier.^8^ Em seguida, enveredou pelo campo geral da Cardiologia e sua procura era tal que associava a localização da dor torácica à artéria coronária acometida, além de estabelecer com eficiência a distribuição espacial dessa circulação. Durante a procura, comentava jocosamente que a obstrução de artérias coronárias também provocaria a ocorrência de um sopro cardíaco no tórax. Mesmo dessa afirmação não reconhecida, acentuava seu exemplo pela diretriz da procura científica incessante.

Descreveu tortuosidades de artérias coronárias como causa de isquemia. Certa vez, propôs provocar o infarto do septo interventricular para diminuir e/ou eliminar a obstrução da via de saída ventricular, direita e/ou esquerda, em miocardiopatia hipertrófica, para intranquilidade de seus pares na ocasião. Hoje em dia, este é um dos procedimentos utilizados nesta doença.

O livro Cardiopatia Isquêmica: Aspectos de Importância Clínica, lançado em 1989 em coautoria com sua esposa Valéria Bezerra de Carvalho, da Editora Sarvier,^9^ mostrou-se de grande informação. Na obra, lançou novos conceitos sobre a dor cardíaca, relacionando a parede acometida com a topografia da dor, e sobre as pressões intracavitárias.

Como pioneiro e para acentuar sua característica de marcas históricas, propôs a realização de um transplante cardíaco em São Paulo, que não foi concretizado por impedimentos legais da época, um ano antes do sul-africano Christian Barnard ter realizado a primeira cirurgia no mundo. Para lembrar o feito, tal cirurgia aconteceu em 3 de dezembro de 1967, às 5h25, na Cidade do Cabo, no hospital Grute-Schuur. Em 1970, sempre de mãos dadas com o progresso, estimulou o cirurgião Euryclides de Jesus Zerbini a realizar a primeira cirurgia de infarto agudo do miocárdio, além de ter se envolvido com as primeiras desobstruções de artérias coronárias, através do uso de laser, em 1976. Neste ínterim, graças ao envolvimento para mais descobertas no campo operatório, o destacado cirurgião Adib Jatene denominava-o como “um dos pais da cirurgia cardíaca no Brasil”. Através da intromissão em outros campos, como clínico perspicaz que era, permitia-se abertamente comentar que “se a cirurgia fosse difícil, seria ela realizada pelos clínicos”, o que a todos descontraía, naquela que era uma afirmação da profícua atuação do clínico. Em 1970, foi também pioneiro na introdução da ecocardiografia em nosso meio, o que facilitou sobremaneira aspectos diagnósticos, principalmente na cardiologia pediátrica, que sofria mudanças consistentes, no âmbito diagnóstico terapêutico, clínico e cirúrgico.

Ademais, participou ativamente na construção do Instituto do Coração, inaugurado em 1978, que se tornou outro apêndice de realce científico do Hospital das Clínicas da Universidade de São Paulo.

Construiu, junto com Luiz Décourt, Fúlvio Pillegi, João Tranchesi, E. J. Zerbini, Geraldo Verginelli, Delmond Bittencourt e Egas Armelin, dentre outros, a fase áurea da Cardiologia e os frutos hoje colhidos por tantos discípulos que se tornaram nomes também de realce, na representatividade atual do próprio Professor Titular da Cardiologia, José Antonio Franchini Ramires.

Na complementação de seu poderio cultural, foi autor do livro que chamou a atenção para a sua capacidade, ao aproximar de fato a Matemática, sua paixão declarada, à Medicina. Esse título, Matemática da Arquitetura Humana-Idiometria Humana-Novos Rumos da Normalidade.^10^ define a tese dos padrões de normalidade para compreender de forma adequada o funcionamento do corpo humano, guiado pelas regras da Matemática. E ele explica que “normal é o que tem que ser, é a verdade a ser atingida: não pode ser um intervalo; é um e somente um número e para obtê-lo é preciso conhecer a variável explicativa, áurea, dominante e, logo, universal e básica”.

É a partir desses conceitos que Macruz explica o significado de normalidade e define o que é normal, para então mostrar o que, quando e onde tratar, levando em conta a variação racial, cultural e alimentar, influenciada pela percepção.

Dono de uma formação sólida, foi um dos maiores clínicos dos nossos tempos. Criou uma enorme escola e deixou discípulos mundo afora, que muito o admiravam e admiram. Esse é o maior legado de um Mestre.

Médico brilhante, incansável em pensar soluções para diferentes cardiopatias, não era um cientista, mas um médico com ótima formação e que se utilizava de conhecimentos em diferentes áreas buscando entender ou tratar doenças cardíacas. Dessa forma, ele envolvia Matemática, Física, Biologia e Filosofia, enxergando o mundo e a Medicina de uma forma ampla e abrangente, sem retirar do médico a responsabilidade de procurar saber cada vez mais do paciente e da doença que o acometia. Nunca se sentiu satisfeito com o que existia e, por esse motivo, brincava ao dizer “a luz chegou”, expondo ideias que muitas vezes pareciam absurdas, mas que ao longo dos anos se tornaram soluções da prática clínica.^11^

Como Décourt, era dono de uma cultura sólida. Literatura, música, arte, tudo fazia parte de seu cotidiano. Não era apenas médico e pesquisador, mas um ser humano com enorme curiosidade. Fomos seus discípulos e sentimos um orgulho definitivo dele. Quando se aposentou, a Faculdade e o InCor perderam um pedaço acadêmico. No entanto, continuávamos em contato e suas ideias persistiam estimulantes.

Suas características pessoais tornaram-no inconfundível e, dessa forma, facilmente sabíamos quando ele estava chegando. Nosso relacionamento era muito mais do que profissional, pois éramos todos muito amigos. Ele terminava nossas conversas, do alto de sua sabedoria, com as palavras “Compreende bem!”, em seu típico timbre gutural. Ou, para começo da conversa, dizia “A luz chegou!”, com grande senso de humor.

Caro amigo, agora, esteja onde estiver, você está às voltas com Décourt e Zerbini, e certamente criando. Você provou que o ser humano é viável. Radi, você nos faz muita falta.

Deixa-nos, assim, um dos mestres mais brilhantes que a cardiologia já produziu, Radi Macruz. Não é fácil descrevê-lo humanamente e como acadêmico. Em ambos os setores, seu brilho era indiscutível. Era dono de uma personalidade complexa, genial, e por isso não era fácil conhecê-lo.

Radi Macruz, sua vida foi pródiga em realizações, decorrentes da lógica, em meio à ética médica e humana, sua colocação “ENTENDEU BEM?” nos fica como a pergunta que remete a procura em prol da verdade e da assertiva. Saudades, muitas, na continuidade de seu estímulo.

Macruz, esteja certo que você marcou gerações que jamais o esquecerão. Toda vez que entrarmos na sala de aula do InCor, denominada Sala Macruz, a luz estará acesa com o brilhantismo dos raios de luz deixados por ti.
