# Supplementary material for: Radi Macruz – O Legado de um Ícone
Source: Arq Bras Cardiol. 2021 Apr 8;116(4):679–81. [Article in Portuguese] doi: 10.36660/abc.20201341 (PMC8121405; doi:10.36660/abc.20201341)
Supplement: Translation

# Translation: Radi Macruz – The Legacy of an Icon

**Affiliations:** 1 São PauloSP Brazil Instituto do Coração do Hospital das Clínicas da Faculdade de Medicina da Universidade de São Paulo, São Paulo, SP - Brazil.

**Keywords:** Radi Macruz, Cardiologists/trends, Faculty, Physicians, Cardiology, Ethics, Medical

Radi Macruz, Associate Professor in the Department of Cardio-Pulmonology at Faculdade de Medicina da Universidade de São Paulo, died in São Paulo on November 30, 2020, at the age of 95.

He was born on July 5, 1925 in São Paulo, son of the Lebanese couple Kalil and Nini Macruz. Attended medical school at FMUSP and became the great pioneer physician, introducing many advances that made him one of the most relevant icons. He was an idealizer in pursuit of the most appropriate dynamics of science and scientific discoveries.

His academic history has always been based on deep knowledge and the progress of cardiology sciences as a whole.

Encouraged by his father, who stressed the importance of doctors in society, he started medical studies at Universidade de São Paulo in 1946, even though Mathematics had been his passion since he was a young man, from his studies in schools in the interior of São Paulo, where he lived with ten more siblings, in addition to the seven cousins, from his paternal uncle.

However, in his professional career, it was Mathematics that influenced his scientific work in Cardiology, interconnected on many levels. This resulted in the creation of a namesake index — the PPR interval in electrocardiography — for the characterization of atrial overloads.^1^^–^^4^

Once, for this creation and so many others, Instituto do Coração (InCor) received one of the greatest electrocardiography authorities in the world, South African Leo Schamroth,^5^^,^^6^ to give a lecture. Escorted by Dr. Charles Mady to the seminar room, Mr. Schamroth met Macruz at the door. When introduced to Macruz, Mr. Schamroth asked if he was the Macruz index one. Mr. Mady replied yes, then hurried to open his book and show the page by commenting on the said index. They both became friends. Macruz did not need any great technologies to show his genius.

He believed that there is no Medicine without Humanism, that the passion of life was the discovery of different things, that critical mass leads to thinking and development, that logic reaches the truth, that the spirit of research is nothing more than questioning itself, that passion unravels the mysteries of nature (Figure 1).

**Figure 1:** Photo of Radi Macruz, in the expression of seriousness and responsibility, resulting from thinking focused on possible scientific discoveries.

Since returning from the United States, where he participated in a study group with his friend Sarnoff,^7^ he has become an inexhaustible source of ideas and scientific production. As he had a very broad general education, he did not limit himself to creating in subspecialties, which was too little for him, as he had a solid background in mathematics and statistics, which helped him a lot.

With all these directions in life, he started to try to understand Pediatric Cardiology, a specialty that involved a lot of diagnostic and therapeutic difficulties, especially in the 1950s and 1980s. In that period, he educated himself with Munir Ebaid, and became one of those responsible for the worldwide recognition of this specialty, as a Brazilian icon.

He innovated with the treatment of pulmonary hypertension in congenital heart diseases, with the opening of an atrial septal communication. He introduced original concepts about the individual normality of blood pressure. He wanted to perform transposition of the great arteries in patients with heart failure, with a normal right ventricle.

He was so knowledgeable that everyone would looking for him to get help with management difficulties. He would then start his medical visits, always with a lot of interested people, as his discussions at the bedside were anthological and highly sought-after. He diagnosed complex cardiopathies at the bedside, with physical examination, simple radiology and electrocardiography, merely based on clinical reasoning. He was one of the very few doctors who were not afraid of exposing their thoughts in a clinical discussion, when people would often find it nonsense and, later, would bow to a shining light.

For this reason and with such motivation when entering the ward at Hospital das Clínicas or Instituto do Coração, he would turn off the lights then say that “the light had arrived”.

No wonder in 1983 he released the first specialty book in Brazil, entitled “*Cardiologia Pediátrica*” (Pediatric Cardiology), with Dr. Rachel Snitcowsky, published by Editora Sarvier.^8^ Then, he entered the general field of Cardiology and his flair for investigation was such that he associated the site of chest pain with the coronary artery affected, in addition to establishing the spatial distribution of this circulation. While doing these investigations, he would jokingly say that the obstruction of coronary arteries would also cause a heart murmur in the chest. Even from this unrecognized statement, his leading role in unceasing scientific search would be further cemented.

He described the tortuosity of coronary arteries as the cause of ischemia. Once, he suggested to provoke an interventricular septal infarction decrease and/or eliminate a right and/or left ventricular outflow tract obstruction into hypertrophic cardiomyopathy, for the uneasiness of his peers at the time. Today, it is one of the procedures used in this disease.

Co-authoring with his wife Valéria Bezerra de Carvalho, he shared valuable information in the book “*Cardiopatia Isquêmica-Aspectos de Importância Clínica*” (Ischemic Heart Disease — Aspects of Clinical Importance), published by Editora Sarvier,^9^ in 1989. In this book, he introduced new concepts about cardiac pain, relating the affected wall to the topography of pain, in addition to new concepts about intracavitary pressures.

As a pioneer and to enhance his typical historical marks, he suggested to conduct a heart transplant surgery in São Paulo, not performed due to legal impediments at the time, one year before the South African Christian Barnard performed the first surgery in the world. The surgery took place on December 3, 1967 at 5.25 am in Cape Town at the Grute-Schuur Hospital. Always hand in hand with progress, he encouraged surgeon Euryclides de Jesus Zerbini to perform the first acute myocardial infarction surgery in 1970, and got involved with the first coronary artery laser unblocking procedures in 1976. In the meantime, due to his involvement in further discoveries in the operative field, the prominent surgeon Adib Jatene called him “one of the fathers of cardiac surgery in Brazil”. His connection with other fields, as an astute general practitioner, allowed him to state openly that “if surgery was a difficult thing, it would be performed by the general practitioners,” which amused everyone, as his statement reaffirmed the brilliant performance of general practitioners. He was also a pioneer in the introduction of echocardiography in Brazil in 1970, which greatly supported diagnosis in pediatric cardiology, which underwent consistent changes, especially in the diagnostic and therapeutic, clinical and surgical fields.

In addition, he actively participated in the construction of Instituto do Coração, opened in 1978, which became another scientific enhancement facility of Hospital das Clínicas da Universidade de São Paulo.

With Luiz Décourt, Fúlvio Pillegi, João Tranchesi, E J Zerbini, Geraldo Verginelli, Delmond Bittencourt, Egas Armelin, and others, he built the golden phase of Cardiology and the fruits today are harvested by so many disciples who have also become names of prominence, such as the Full Professor of Cardiology Jose Antonio Franchini Ramires.

Complementing his cultural power, he wrote a book that drew attention to the characterization of his capacity, when he actually connected Mathematics, his declared passion, to Medicine. The title “*Matemática da Arquitetura Humana – Idiometria Humana – Novos Rumos da Normalidade*” (Mathematics of Human Architecture — Human Idiometry — New Directions of Normality),^10^ presents the thesis of normality patterns and outlines the functioning of the human body guided by the rules of Mathematics. And he explains that “normal is what something has to be, it is the truth to be reached: it cannot be an interval; it is one and only one number and, to obtain it, one must know the golden dominant explanatory, therefore universal and basic variable.”

Based on these concepts, Macruz explains what normality means, defines what is normal, then shows what to treat, when to treat and where to treat, considering racial, cultural and dietary variations influenced by perception.

Of a solid background, he was one of the greatest general practitioners of our times. He set trends and had disciples around the world, who admired and still admire him greatly. This is the greatest legacy that a Master can leave.

A brilliant doctor, tireless thinker of solutions for different heart diseases, he was not a scientist, but a doctor with an excellent background, who used knowledge from different areas in order to understand or treat heart diseases. As such, he mixed up mathematics, physics, biology and philosophy, seeing the world and medicine in a broad and comprehensive way, without denying doctors’ responsibility for getting more and more information about their patients and their disease. He was never happy with whatever was available and, for this reason, he would jokingly announce “the light has arrived,” sharing ideas that often seemed absurd, but over the years have come to exist as solutions in clinical practice.^11^

Like Décourt, he had a solid cultural background. Literature, music, art, everything was part of his daily life. He was not only a doctor and a researcher, but a human being with enormous curiosity. We were his disciples, and we are definitely proud of him. When he retired, the Medical School and Incor lost an academic piece. However, we kept in touch and his ideas continued to flow.

His personal characteristics made him unmistakable, so we would easily know when he was coming in. Our relationship was much more than professional, as we were all very close friends, and he ended our conversations, from the height of his wisdom, with the words “got it?”, with his typical deep voice. Or, at the beginning of our conversations, he would say “the light has arrived,” with a great sense of humor.

Dear friend, wherever you are, now you are around Décourt and Zerbini, and certainly creating. You have proved that the human being is viable. Radi, we miss you terribly.

One of the most brilliant masters that cardiology has ever produced, Radi Macruz, leaves us all. It is not easy to describe him humanly, and as an academic. In both sectors, his brilliance was indisputable. He had a complex and brilliant personality, so it was not easy to know him well.

Radi Macruz, your life was full of achievements, resulting from logic, in the midst of medical and human ethics, and your saying “GOT IT?” is left as a question in the pursuit of truth and assertion. We miss you as we all know that this longing is a source of encouragement.

Macruz, be sure that you have marked generations that will never forget you and every time we enter InCor’s classroom, called the Macruz Room, the light will be on with the brilliance of rays of light, left by you.
